# Phase angle identifies a muscle quality phenotype associated with physical performance in U.S. marines

**DOI:** 10.3389/fphys.2026.1904857

**Published:** 2026-07-28

**Authors:** Adam W. Potter, Christy L. Christopoulos, Karl E. Friedl

**Affiliations:** 1 U.S. Army Research Institute of Environmental Medicine, Natick, MA, United States; 2 Training and Standards Division, Training and Education Command, United States Marine Corps, Quantico, VA, United States

**Keywords:** bioelectrical impedance analysis, body composition, military personnel, muscle quality, performance efficiency, phase angle, physical performance, physical readiness

## Abstract

**Objective:**

To evaluate whether phase angle (PhA) provides physiological information beyond DXA-derived body composition for explaining physical performance in active-duty U.S. Marines.

**Methods:**

We analyzed data from 2,177 active-duty U.S. Marines with bioelectrical impedance analysis, dual-energy X-ray absorptiometry (DXA), and standardized physical performance testing. Phase angle was expressed in degrees and standardized within sex to define Phase Angle Muscle Quality (PhA-MQ) quartiles. Outcomes included Physical Fitness Test (PFT), Combat Fitness Test (CFT), estimated VO_2_max, and performance normalized to lean mass. Multivariable linear regression models adjusted for DXA-derived fat-free mass index (FFMI), percent body fat (%BF), age, and sex were used to assess independent associations. Incremental explanatory value was quantified using change in R^2^ (ΔR^2^) and partial R^2^. Nonlinear associations were examined using spline models.

**Results:**

Physical performance increased progressively across PhA-MQ quartiles, with the greatest separation observed for lean-mass–normalized outcomes. Across PhA quartiles, mean PFT scores increased from 236 to 271 points; while CFT scores increased from 240 to 272 points. In adjusted models, PhA remained independently associated with PFT (ΔR^2^ = 0.013, p < 0.001) and CFT (ΔR^2^ = 0.006, p<0.001). Stronger effects were observed for performance efficiency measures, including PFT per kg lean mass (ΔR^2^ = 0.020; partial R^2^ = 0.055) and CFT per kg lean mass (ΔR^2^ = 0.012; partial R^2^ = 0.051). In contrast, associations with VO_2_max were minimal and not statistically significant (p = 0.088). Joint models demonstrated that PhA retained independent explanatory value alongside FFMI and %BF, particularly for lean-mass–normalized outcomes. Continuous spline analyses supported monotonic relationships between PhA and performance.

**Conclusions:**

Phase angle identifies a physiological phenotype associated with enhanced physical performance independent of DXA-derived body composition. These findings support phase angle as a marker of muscle quality-related physiological status rather than a direct measure of muscle quality.

## 1 Introduction

Military organizations require body-composition metrics that are practical at scale yet still relevant to health, physical performance, and operational readiness. In that context, conventional anthropometric and imaging-derived measures remain important, but they do not fully resolve why individuals with similar body size or lean mass can differ substantially in functional capacity. Recent reviews have emphasized that the relationship between body composition and readiness is more complex than body mass or fatness alone, particularly when the outcomes of interest involve repeated high-intensity efforts, load carriage, maneuverability, and fatigue resistance (McGregor et al., 2014; Naimo et al., 2021).

One reason for this gap is that muscle quantity and muscle quality are not synonymous. In younger and occupationally active populations, muscle quality is increasingly understood as the functional capacity of muscle tissue relative to its size, encompassing not only force or power per unit mass but also structural and compositional features that influence performance. These include intracellular versus extracellular fluid distribution, cellular integrity, contractile efficiency, and tissue composition. Individuals with similar amounts of lean tissue may still differ substantially in the functional capacity of that tissue and in the performance generated per unit muscle mass. This distinction is particularly relevant in physically demanding populations where operational performance depends not only on tissue quantity, but on the physiological quality and functional efficiency of that tissue. Thus, a person may have substantial lean mass yet still differ meaningfully in the quality of that tissue and in the performance delivered by it (Di Vincenzo et al., 2021; Naimo et al., 2021; Akamatsu et al., 2022; da Costa Pereira et al., 2024).

Phase angle (PhA), derived directly from bioelectrical impedance resistance and reactance, has emerged as a candidate physiological marker associated with muscle quality and functional performance. Mechanistically, PhA reflects the electrical behavior of tissues and is influenced by membrane capacitance, body cell mass, and fluid distribution. Higher values are generally interpreted as indicating better cellular integrity and a more favorable intracellular-to-extracellular milieu, whereas lower values have been associated with impaired nutritional and functional status. Although the clinical literature first established this relevance in disease and aging, more recent work has extended interest in PhA to the assessment of muscle function and muscle quality more broadly (Norman et al., 2012; Lukaski and Talluri, 2023; Ward and Brantlov, 2023).

That extension is supported by a growing empirical literature. PhA is lower in sarcopenia, correlates with muscle-quality indices, and has been associated with muscle strength, aerobic fitness, and sport performance across multiple populations. In athletes and physically active adults, higher PhA has been linked with handgrip strength, jump performance, and running performance, while resistance training and habitual exercise appear capable of increasing PhA over time. However, this literature is still heterogeneous, and most data come from clinical cohorts, athletes, or general healthy populations rather than military personnel performing service-specific fitness tasks (Di Vincenzo et al., 2019; Genton et al., 2020; Di Vincenzo et al., 2021; Akamatsu et al., 2022; Martins et al., 2022; Yamada et al., 2022; Cirillo et al., 2023; Sardinha and Rosa, 2023; Varanoske et al., 2023; da Costa Pereira et al., 2024).

This gap is particularly relevant because multifrequency bioelectrical impedance has become increasingly feasible for use in physically active and military-age populations (Potter et al., 2022b; Looney et al., 2024). Contemporary systems demonstrate high repeatability and acceptable field utility, while offering practical advantages over methods such as DXA in operational settings (Potter et al., 2025). Yet an unresolved question is whether PhA contributes information that is independent of standard body-composition metrics such as DXA-derived fat-free mass index (FFMI) and percentage body fat (%BF), or whether it simply restates the same construct in another form. The present study addresses that question in U.S. Marines by testing whether PhA is associated with physical performance after accounting for DXA FFMI, DXA %BF, age, and sex. In this manuscript, PhA-MQ is used as a shorthand for a phase angle-defined muscle quality phenotype, not as a direct histological or imaging measure of muscle quality. We hypothesized that PhA-MQ would provide independent explanatory value, particularly for performance outcomes that reflect the efficiency or functional quality of available lean tissue rather than tissue quantity alone (McGregor et al., 2014; Campa et al., 2022; Potter et al., 2022b; Looney et al., 2024).

Importantly, throughout this manuscript PhA-MQ refers to a phase angle-defined physiological phenotype associated with muscle quality in prior literature. The term is not intended to imply that phase angle directly measures muscle architecture, intramuscular fat, contractile properties, or histological muscle quality. Rather, it provides a conceptual framework for examining whether phase angle captures physiological characteristics relevant to physical performance beyond conventional body composition metrics.

## 2 Methods

### 2.1 Study population

Data were analyzed from 2,177 active-duty U.S. Marines (1,437 men, 736 women) who underwent concurrent assessment of bioelectrical impedance, dual-energy X-ray absorptiometry (DXA) body composition, and standardized physical performance testing (Potter et al., 2022a). Participants were instructed to be normally hydrated, avoid strenuous exercise for 12 hours, and were assessed in a standardized supine position. All participants wore the standard Marine Corps “green on green” physical training shorts and t-shirts. Participants were evaluated as part of routine operational or research-based assessments. Analyses were conducted using complete-case methods, with individuals included in specific models based on the availability of relevant variables.

#### 2.1.1 Ethics statement

Prior to study activities, all participants provided written informed consent, and women were provided a rapid pregnancy test to establish the absence of detectable pregnancy. Study approval was granted by the US Army Medical Research and Development Command (Fort Detrick, Maryland) and US Marine Corps (Quantico, Virginia) Institutional Review Boards, protocol M10873, approved March 2021. All procedures were conducted in accordance with the ethical standards of the responsible institutional and national research committees and with the Declaration of Helsinki.

### 2.2 Measurements

Body composition was assessed using DXA (iDXA, GE Lunar, Madison, Wisconsin, USA) and fat-free mass index (FFMI), calculated as lean mass divided by height squared (kg/m²), percent body fat (%BF), and total lean mass (kg). FFMI and %BF were included together to distinguish lean tissue quantity from adiposity burden, allowing assessment of whether PhA contributes information beyond both structural and compositional body-size domains.

Following the DXA scan, individuals remained supine on the non-conductive surface, and total body resistance was measured at 50 kHz between the left hand and left foot using a whole-body impedance device (Quantum IV, RJL Systems, Clinton Township, Michigan, USA). Electrodes were placed at the left wrist on a line bisecting the ulnar head and base of the middle finger, and on the left ankle on a line bisecting the medial malleolus and base of the middle toe. Phase angle was derived from bioelectrical impedance analysis (BIA) and expressed in degrees. It represents the arctangent of reactance to resistance and reflects the electrical properties of biological tissues, including cell membrane integrity and intracellular water distribution. This raw PhA, in degrees, is the PhA-MQ metric used throughout this manuscript. Body fat (%BF) and FFM were also calculated from the 50 kHz resistance value (Sun et al., 2003).

Physical performance was assessed using standardized U.S. Marine Corps fitness tests. The Physical Fitness Test (PFT) is a composite score derived from pull-ups or push-ups, abdominal crunches or plank, and a 3-mile run, reflecting aerobic capacity and muscular endurance. The Combat Fitness Test (CFT) is a composite score based on movement-to-contact, ammunition lift, and maneuver-under-fire tasks, reflecting strength, power, and combat-relevant performance. Aerobic capacity was estimated using Marine Corps PFT performance and the standardized prediction equation employed within U.S. military research and fitness evaluations (Mello et al., 1988; Potter et al., 2023; Potter et al., 2026). To evaluate muscle functional efficiency, performance outcomes were normalized to lean mass, including PFT per kilogram of lean mass and CFT per kilogram of lean mass.

### 2.3 PhA-MQ phenotype

Phase angle muscle quality (PhA-MQ) was standardized within sex using z-score transformation to account for physiological differences between men and women. Participants were then grouped into sex-standardized quartiles (Q1–Q4), with Q1 representing the lowest and Q4 the highest PhA-MQ values. These quartiles were used to characterize phenotype differences and to evaluate gradients in performance across increasing levels of muscle quality.

### 2.4 Statistical analysis

All analyses were conducted in R (version 4.4.3; R Foundation for Statistical Computing, Vienna, Austria). Raw PhA (degrees) was used in regression analyses, while sex-standardized PhA quartiles were used to define phenotype groups. Continuous associations between PhA and performance outcomes were evaluated using multivariable linear regression models adjusted for DXA-derived fat-free mass index (FFMI), percent body fat (%BF), age, and sex. The independent contribution of PhA was quantified using the change in coefficient of determination (ΔR²) between nested models and partial R² to estimate the proportion of variance uniquely explained by phase angle. To assess whether PhA provided information beyond traditional body composition measures, joint models including PhA, FFMI, and %BF were constructed. Residual modeling was performed to further isolate the contribution of phase angle by first regressing performance outcomes on FFMI, %BF, age, and sex, and then evaluating associations between phase angle and the resulting residuals. Non-linear relationships were assessed using natural spline models to characterize continuous associations between PhA and outcomes. Interaction terms were evaluated to assess potential effect modification by sex. Statistical significance was defined as a two-sided p-value <0.05. Variance inflation factors (VIFs) were calculated to assess multicollinearity among predictors. Model assumptions were evaluated through inspection of residual-versus-fitted plots, normal Q-Q plots, and residual distributions.

## 3 Results

### 3.1 Participant characteristics

The analytic sample included 2,177 active-duty U.S. Marines with high completeness for phase angle and DXA-derived body composition measures. Participant characteristics stratified by Phase Angle Muscle Quality (PhA-MQ) quartiles are presented in **Table 1**. Mean age and body composition profiles were consistent with a physically active military population. Sex-standardization of PhA-MQ reduced but did not eliminate sex-related distributional differences, so sex was retained as a covariate in all models. Mean PFT scores increased from 236 to 271 points across quartiles; while CFT scores increased from 240 to 272 points. Performance normalized to lean mass likewise increased progressively from 4.24 to 4.72 (PFT/kg lean mass) and from 4.33 to 4.78 (CFT/kg lean mass).

**Table 1:** Participant characteristics by PhA-MQ quartiles.

Characteristic	Q1 (lowest)	Q2	Q3	Q4 (highest)
N	545	544	544	544
Age (years), mean	26.2	26.7	26.6	26.4
Male (%)	50.1	69.1	67.5	78.1
Phase angle (°), mean	6.29	7.02	7.48	8.15
DXA Lean Mass (kg), mean	55.6	59.2	60.3	62.9
DXA Fat Mass (kg), mean	18.8	17.1	15.4	13.5
DXA FFMI (kg/m²), mean	18.3	19.5	20.2	21.4
DXA % Body Fat, mean	26.1	23.8	21.2	18.4
PFT score, mean	236	250	258	271
CFT score, mean	240	252	260	272
VO_2_max (mL·kg^-^¹·min^-^¹), mean	41.3	42.6	43.1	44.2
PFT per kg lean mass	4.24	4.39	4.51	4.72
CFT per kg lean mass	4.33	4.45	4.57	4.78

Interpretation: Performance and performance efficiency increased progressively across PhA-MQ quartiles, while changes in FFMI and %BF were modest, supporting a muscle-quality interpretation.

### 3.2 PhA-MQ quartile phenotype

**Figure 1:**
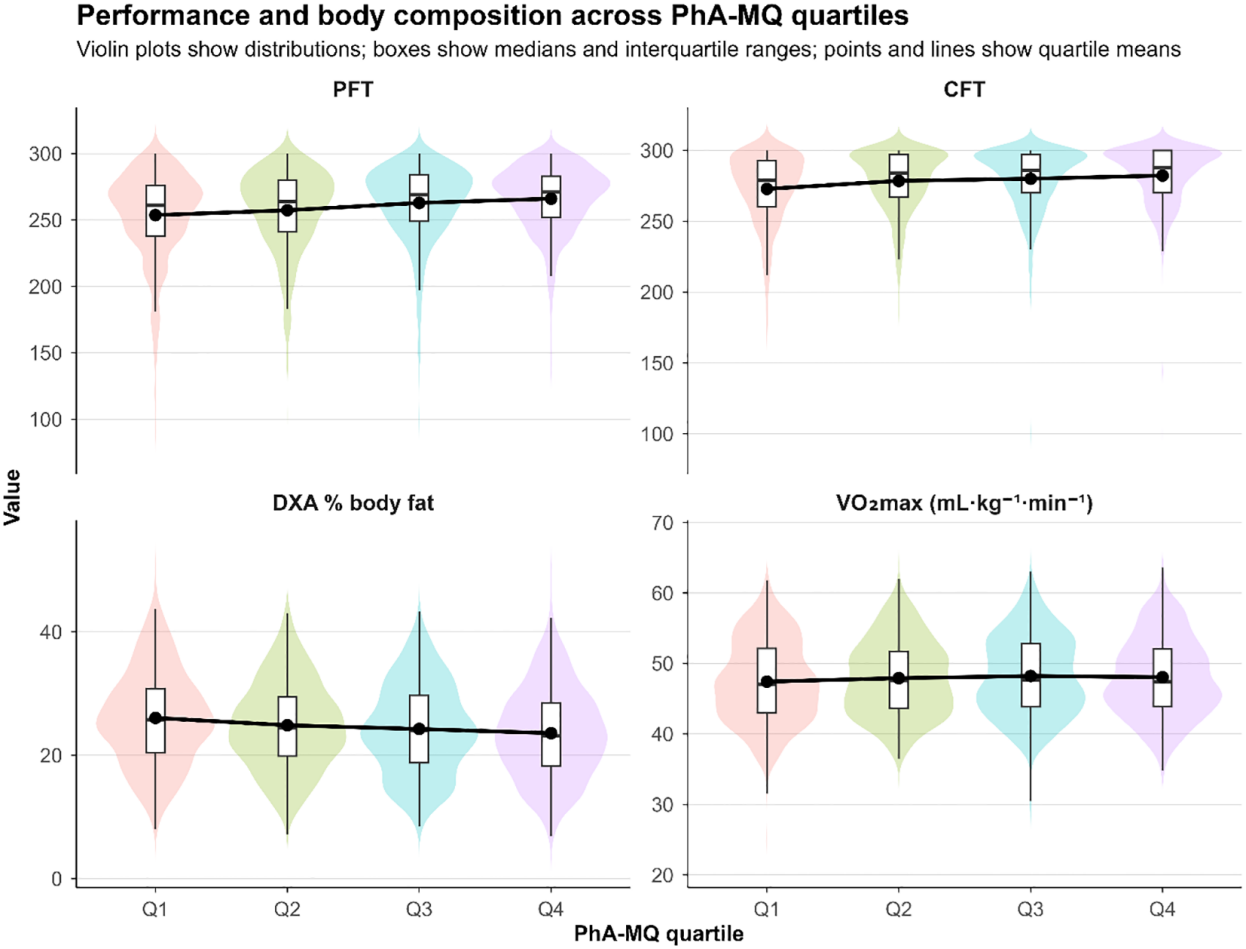
Performance and body composition across Phase Angle Muscle Quality (PhA-MQ) quartiles. Violin plots show the distribution of values within each quartile, boxplots show the median and interquartile range, and overlaid points and lines show quartile means. Higher PhA-MQ quartiles were associated with progressively higher PFT and CFT scores and lower DXA percent body fat, consistent with a more favorable physiological performance phenotype.

Physical performance increased progressively across PhA-MQ quartiles (**Table 1**; **Figure 1**). Both PFT and CFT scores demonstrated monotonic increases from the lowest to highest quartile, indicating a graded relationship between PhA and performance. Performance normalized to lean mass showed even more pronounced separation across quartiles. Both PFT/kg lean mass and CFT/kg lean mass increased consistently, indicating greater performance efficiency at higher PhA-MQ levels (**Figure 1**). We define performance efficiency as performance relative to available lean tissue mass, not metabolic efficiency.

Body composition also differed meaningfully across PhA-MQ quartiles. FFMI increased progressively whereas %BF decreased, indicating that participants with higher PhA generally exhibited more favorable body composition profiles. Importantly, however, these body composition differences did not fully account for the observed performance gradients after multivariable adjustment, indicating that PhA contributed information beyond DXA-derived measures alone.

### 3.3 Independent and incremental associations of phase angle

The independent and incremental contribution of PhA is summarized in **Table 2**, which represents the central quantitative finding of the study. After adjustment for FFMI, percent body fat, age, and sex, PhA remained significantly associated with performance outcomes (**Table 2**). Regression coefficients with corresponding 95% confidence intervals are also presented in **Table 2**. The addition of PhA increased model explanatory power for PFT (ΔR² = 0.013) and CFT (ΔR² = 0.006), indicating that it provides non-redundant information beyond traditional body composition metrics. Importantly, the magnitude of these effects was substantially greater for lean-mass–normalized outcomes, where PhA demonstrated its strongest associations. For PFT per kg lean mass and CFT per kg lean mass, ΔR² values reached 0.020 and 0.012, respectively, with partial R² values exceeding 0.05. These findings indicate that PhA is most strongly related to performance efficiency, rather than absolute performance. In contrast, associations with VO2max were minimal and not statistically significant (ΔR² ≈ 0.001; p = 0.088), suggesting that PhA is less strongly linked to aerobic capacity than to strength- and power-related performance domains. These relationships are further illustrated in **Supplementary Figure 1**, which shows the incremental and independent contributions of phase angle across outcomes.

**Table 2:** Multivariable associations of phase angle with performance outcomes.

Outcome	N	R² (base)	R² (+PhA)	ΔR²	β (95% CI)	SE	p-value	Partial R²
PFT	2097	0.306	0.319	0.0130	5.34 (3.68, 6.99)	0.84	<0.001	0.0188
CFT	2064	0.234	0.240	0.0063	2.81(1.48, 4.15)	0.68	<0.001	0.0082
VO_2_max	2041	0.480	0.481	0.0007	0.24 (-0.04, 0.51)	0.14	0.088	0.0014
PFT per kg lean	2097	0.644	0.664	0.0195	0.105 (0.086, 0.123)	0.0095	<0.001	0.0549
CFT per kg lean	2064	0.759	0.772	0.0123	0.086 (0.070, 0.102)	0.0082	<0.001	0.0510

(Adjusted for DXA FFMI, %BF, age, and sex).

Interpretation: Phase angle remained independently associated with performance, with the strongest effects observed for lean-mass-normalized outcomes.

### 3.4 Relative contributions in joint models

To further clarify the role of PhA relative to body composition, **Table 3** presents joint models including PhA, FFMI, and percent body fat simultaneously. These models demonstrate that PhA retains independent explanatory value across outcomes, even when accounting for both structural (FFMI) and adiposity (%BF) components. Importantly, the magnitude of partial R2 for PhA (~0,05 for normalized outcomes) approaches that of traditional body composition variables, supporting its relevance as a complementary physiological signal rather than a trivial covariate. Although the incremental variance explained by PhA was modest for absolute performance outcomes, it remained statistically significant despite adjustment for robust DXA-derived body composition variables. The greatest independent contribution was consistently observed for lean-mass-normalized performance outcomes. In contrast, for VO2max, phase angle contributes minimally relative to body composition variables, reinforcing a domain-specific interpretation. **Tables 2**, **3** demonstrate that PhA captures a distinct physiological dimension that is not reducible to muscle mass or adiposity, with its strongest influence observed in measures of performance relative to tissue mass. Collectively, these findings suggest that PhA may be more closely related to integrated muscular performance characteristics and tissue functional status than to aerobic capacity alone.

**Table 3:** Joint models including phase angle, FFMI, and % body fat.

Outcome	N	R²	β (phase) (95% CI)	p (phase)	Partial R² (phase)	β (FFMI) (95% CI)	p (FFMI)	Partial R² (FFMI)	β (%BF) (95% CI)	p (%BF)	Partial R² (%BF)
PFT	2097	0.319	5.34 (3.68, 6.99)	<0.001	0.0188	6.12 (4.23, 8.01)	<0.001	0.072	-2.45 (-3.29, -1.61)	<0.001	0.041
CFT	2064	0.240	2.81 (1.48, 4.15)	<0.001	0.0082	4.88 (3.26, 6.50)	<0.001	0.061	-1.92 (-2.61, -1.23)	<0.001	0.033
VO_2_max	2041	0.481	0.24 (-0.04, 0.51)	0.088	0.0014	0.89 (0.58, 1.20)	<0.001	0.052	-0.67 (-0.91, -0.43)	<0.001	0.048
PFT per kg lean	2097	0.664	0.105 (0.086, 0.123)	<0.001	0.0549	0.082 (0.057, 0.107)	<0.001	0.031	-0.051 (-0.069, -0.033)	<0.001	0.028
CFT per kg lean	2064	0.772	0.086 (0.070, 0.102)	<0.001	0.0510	0.061 (0.041, 0.081)	<0.001	0.025	-0.043 (-0.059, -0.027)	<0.001	0.024

Interpretation: Phase angle retained independent explanatory value alongside FFMI and %BF, particularly for lean-mass-normalized performance.

### 3.5 Residual performance

Residual modeling further supports this interpretation. After removing variance attributable to FFMI, percent body fat, age, and sex, phase angle remained significantly associated with residual PFT and CFT (**Supplementary Table 1**). Although effect sizes were modest, the persistence of these associations confirms that phase angle reflects an independent physiological signal beyond body composition. Variance inflation factors were uniformly low, indicating that multicollinearity did not materially influence the regression models (**Supplementary Table 3**).

### 3.6 Continuous associations (spline models)

**Figure 2:**
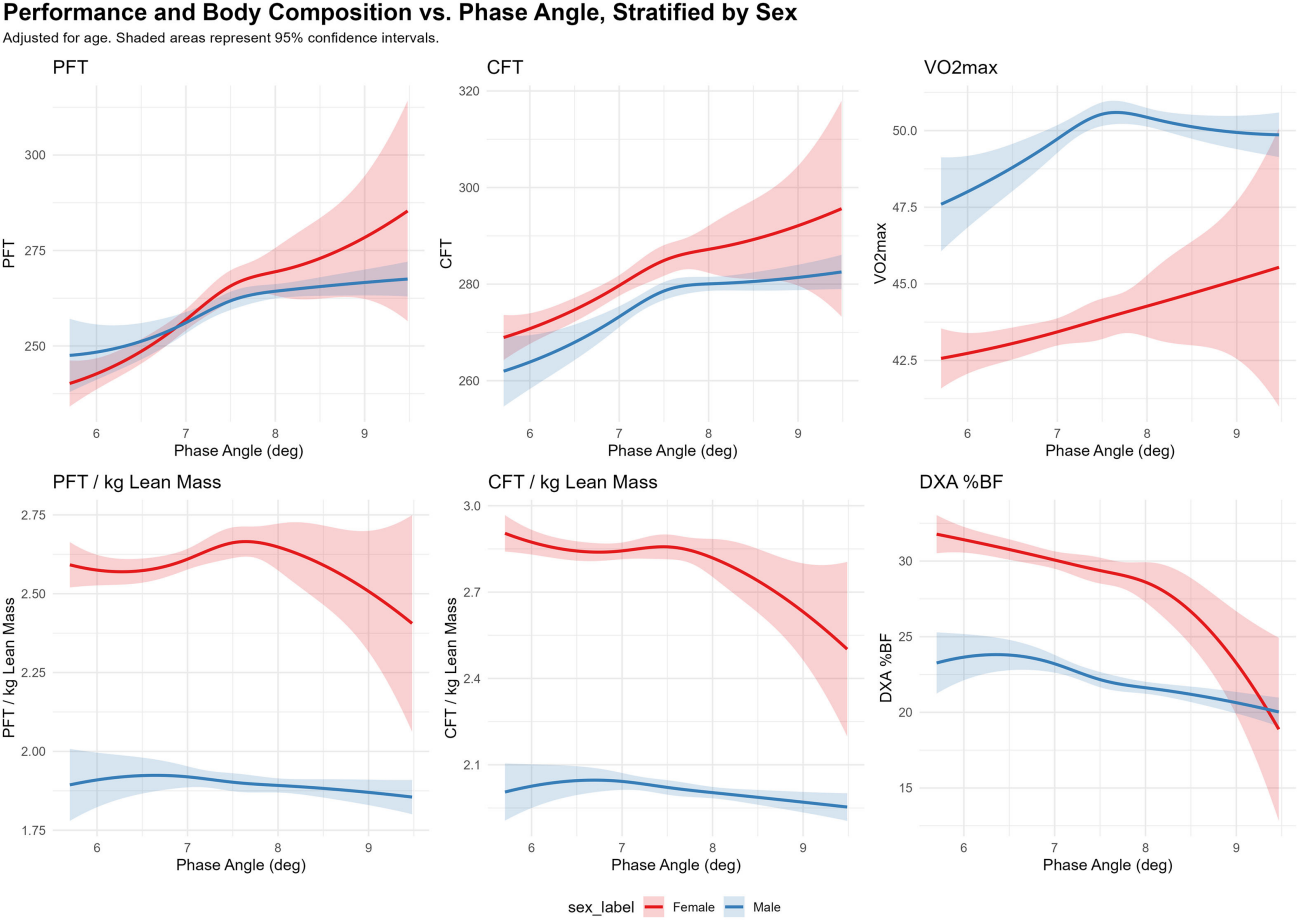
Spline models of phase angle muscle quality (PhA-MQ) and performance outcomes. Six-panel figure showing continuous associations between phase angle and PFT, CFT, VO_2_max, PFT/kg lean, CFT/kg lean, and percent body fat.

Spline models demonstrated continuous, approximately monotonic relationships between phase angle and physical performance outcomes (**Figure 2**). Higher PhA values were associated with progressively greater PFT and CFT scores, as well as improved performance per unit lean mass. These relationships were evident across the observed range of PhA values and were not driven by threshold effects. Sex interaction terms were not statistically significant, supporting pooled analyses with adjustment for sex. Collectively, these results support a consistent, continuous association between PhA and physical performance across the population. Specific data for these spline models is available in **Supplementary Table 2**.

## 4 Discussion

The principal finding of this study is that phase angle provided non-redundant explanatory information for Marine physical performance beyond DXA-derived FFMI, DXA %BF, age, and sex. The added value was most evident for the PFT and CFT outcomes, and especially for the same tests expressed relative to lean mass. This pattern argues against viewing PhA-MQ as merely another proxy for body size or lean tissue quantity. Instead, the present results support the interpretation that PhA-MQ captures a physiological dimension associated with physical performance that is not fully represented by DXA-derived body composition (**Tables 2**, **3**) (Di Vincenzo et al., 2019; Genton et al., 2020; Akamatsu et al., 2022; Martins et al., 2022; Cirillo et al., 2023; da Costa Pereira et al., 2024).

That interpretation is biologically plausible. DXA quantifies tissue compartments well, but it does not directly resolve membrane function, intracellular hydration, or other qualitative properties of muscle tissue that may influence force production and movement economy. By contrast, PhA is a raw impedance-derived variable tied to the electrical behavior of tissues and is influenced by the balance between conductive and capacitive properties of the body. This likely explains why PhA has repeatedly been associated with sarcopenia, muscle function, and muscle-quality constructs even when simple mass measures do not tell the full story. At the same time, the current data should not be interpreted as evidence that PhA directly measures histological muscle quality; rather, it is better described as an integrated marker of performance-relevant muscle tissue status (Norman et al., 2012; Di Vincenzo et al., 2021; Akamatsu et al., 2022; Lukaski and Talluri, 2023; Ward and Brantlov, 2023; da Costa Pereira et al., 2024). Importantly, these findings should not be interpreted as demonstrating that PhA directly measures muscle quality. Rather, our results support PhA as an integrated physiological marker associated with tissue function and physical performance, consistent with recent systematic reviews.

The stronger associations with PFT and CFT performance than with estimated VO2max are also informative. The Marine fitness tests are not pure physiological isolates; they reward repeated force generation, body-mass management, movement economy, and the ability to translate available lean tissue into useful performance. Those features align more closely with the concept of muscle quality than with cardiorespiratory capacity alone. This interpretation is consistent with prior reviews showing that PhA is related more consistently to muscle strength and power than to aerobic indices, and with sport-specific data linking PhA to running, jump, and other field-based performance outcomes. The current findings therefore suggest that in Marines, PhA may be most useful as a marker of performance efficiency rather than as a marker of aerobic fitness per se (Di Vincenzo et al., 2019; Genton et al., 2020; Naimo et al., 2021; Martins et al., 2022; Cirillo et al., 2023; Varanoske et al., 2023).

This distinction has practical implications for military medicine and human performance monitoring. In field settings, DXA is informative but resource-intensive, whereas modern multifrequency BIA platforms are faster, portable, and scalable. If PhA can identify Marines who produce better PFT/CFT performance for a given amount of lean tissue, it could serve as a useful adjunct for readiness monitoring, training evaluation, and potentially for identifying individuals whose functional status is out of proportion to their size-based body-composition profile. The translational message, however, should remain disciplined: PhA is not a replacement for performance testing, nor does it supersede DXA. Its value lies in complementing those tools by providing an accessible indicator of tissue functional status (McGregor et al., 2014; Campa et al., 2022; Potter et al., 2022b; Looney et al., 2024; Potter et al., 2025). Although ΔR^2^ values were modeset, these improvements were observed after adjustment for highly informative DXA-derived measures. Consequently, the additional explanatory value attributed to PhA represents non-redundant physiological information obtained from a rapid, portable, and inexpensive assessment.

The literature also supports the possibility that PhA may be responsive to training adaptation. Resistance training appears capable of increasing PhA, and people with regular exercise habits tend to have higher values than those without such habits. This is relevant to Marines because improvements in readiness depend not just on maintaining lean mass but on improving the functional characteristics of that tissue under repeated training exposures. Accordingly, one of the most important implications of the present findings is longitudinal: future work should determine whether changes in PhA track changes in PFT/CFT performance, recovery from high training loads, or resilience during sustained operational stress (Yamada et al., 2022; Sardinha and Rosa, 2023).

These findings also support the evolving emphasis within military human performance programs on assessing functional readiness rather than relying exclusively on static measures of body composition. While DXA quantifies tissue quantity, phase angle appears to capture complementary physiological information related to how effectively that tissue contributes to physical performance. Accordingly, phase angle may have value as an adjunctive readiness assessment in military populations where scalable field-based tools are required.

There are some limitations inherent to this study. Hydration status influences impedance measures but the persistence of associations after adjustment for body composition and the consistency across outcomes suggest that PhA captures more than acute fluid variation alone. The weaker findings for VO2max should be interpreted cautiously because estimated values are less physiologically specific than directly measured gas-exchange data. Although phase angle has been widely described as a surrogate marker of muscle quality, this study did not include direct measurements of muscle architecture, intramuscular adipose tissue, specific force, or histological muscle characteristics. Therefore, the present findings should be interpreted as supporting a physiological phenotype associated with muscle quality rather than direct validation of muscle quality itself. Lastly, findings from Marines may not generalize directly to less fit military populations or to civilian cohorts (Norman et al., 2012; Campa et al., 2022; Potter et al., 2022b; Lukaski and Talluri, 2023; Ward and Brantlov, 2023; Looney et al., 2024).

These findings refine how phase angle should be positioned in this manuscript. The most defensible conclusion is not that PhA is a better body-composition metric than DXA FFMI or %BF, but that it appears to capture an additional dimension of muscle-related functional quality that helps explain why some Marines perform better than others despite similar body composition. That argument is strongest for PFT/CFT outcomes and strongest still when performance is expressed relative to lean mass. Framed this way, the study contributes a clinically and operationally relevant message: readily obtainable bioelectrical data may help move military performance assessment beyond tissue quantity alone and toward the functional value of that tissue (McGregor et al., 2014; Akamatsu et al., 2022; Potter et al., 2022b; Sardinha and Rosa, 2023; da Costa Pereira et al., 2024; Looney et al., 2024). More broadly, these findings support continued evolution of military body composition paradigms beyond static assessments of tissue quantity alone toward physiologically relevant indicators of functional readiness and performance capacity.

## 5 Conclusion

Phase angle provides physiological information beyond DXA-derived body composition that helps explain physical performance in U.S. Marines. Its strongest associations were observed for lean-mass-normalized performance outcomes, suggesting that phase angle reflects a physiological phenotype related to performance efficiency rather than simply tissue quantity. These findings support phase angle as a practical adjunct to conventional body composition assessment for evaluating physical readiness in military populations.

## Data Availability

The raw data supporting the conclusions of this article will be made available by the authors, without undue reservation.

## Author disclaimer

The opinions or assertions contained herein are the private views of the author(s) and are not to be construed as official or reflecting the views of the Army, Marine Corps, or Department of Defense. Any citations of commercial organizations and trade names in this report do not constitute an official Department of the Army endorsement of approval of the products or services of these organizations. The investigators have adhered to the policies for protection of human participants as prescribed in DODI 3216.02, and the research was conducted in adherence with the provisions of 32 CFR Part 219.
