# Supplementary material for: Phase angle identifies a muscle quality phenotype associated with physical performance in U.S. marines
Source: Front Physiol. 2026 Jul 28;17:1904857. doi: 10.3389/fphys.2026.1904857 (PMC13459097; doi:10.3389/fphys.2026.1904857)
Supplement: Supplementary file 1 [file DataSheet1.docx]

**Graphical Abstract**

**Graphical Abstract.** Conceptual model of Phase Angle Muscle Quality (PhA-MQ) as a dynamic marker of physiological readiness. Lower phase angle is associated with physiological stressors (e.g., energy deficit, illness, overtraining), reflecting reduced cellular integrity and intracellular hydration, and corresponding to decreased performance capacity and increased injury risk. Higher phase angle is associated with training adaptation (e.g., resistance training, improved cellular hydration and metabolism), reflecting enhanced muscle functional status and improved strength and performance. Across this continuum, phase angle is proposed to capture a physiologically relevant dimension of muscle quality beyond tissue quantity alone, linking cellular-level properties to whole-body performance outcomes.
