# Supplementary material for: Phase angle identifies a muscle quality phenotype associated with physical performance in U.S. marines
Source: Front Physiol. 2026 Jul 28;17:1904857. doi: 10.3389/fphys.2026.1904857 (PMC13459097; doi:10.3389/fphys.2026.1904857)
Supplement: Supplementary file 2 [file Supplementaryfile1.docx]

**Supplementary Table S1. Residual Performance Models.** Performance residualized for FFMI, %BF, age, and sex.

| Outcome | N | β (Phase) | SE | p-value | R² |
| --- | --- | --- | --- | --- | --- |
| PFT (residual) | 2097 | 4.88 | 0.81 | <0.001 | 0.021 |
| CFT (residual) | 2064 | 2.36 | 0.66 | <0.001 | 0.012 |
| PFT per kg lean (residual) | 2097 | 0.098 | 0.009 | <0.001 | 0.057 |
| CFT per kg lean (residual) | 2064 | 0.079 | 0.008 | <0.001 | 0.052 |
| VO_2_max (residual) | 2041 | 0.21 | 0.13 | 0.094 | 0.002 |

**Interpretation:** Phase angle remained associated with performance after removing variance attributable to body composition.

**Supplementary Table S2. Spline Model Summary**

| Outcome | N | R² | p (Phase) | p (Interaction) |
| --- | --- | --- | --- | --- |
| PFT | 2097 | 0.32 | <0.001 | 0.18 |
| CFT | 2064 | 0.24 | <0.001 | 0.21 |
| VO_2_max | 2041 | 0.48 | 0.07 | 0.34 |
| PFT per kg lean | 2097 | 0.66 | <0.001 | 0.12 |
| CFT per kg lean | 2064 | 0.77 | <0.001 | 0.15 |
| % Body Fat | 2169 | 0.29 | <0.001 | 0.19 |

**Supplementary Table S3. Linear model diagnostics**

| Outcome | Complete Cases | Model R² | Maximum VIF | Shapiro–Wilk P | Maximum Cook's Distance |
| --- | --- | --- | --- | --- | --- |
| PFT | 2097 | 0.319 | 2.37 | <0.001 | 0.019 |
| CFT | 2064 | 0.240 | 2.39 | <0.001 | 0.078 |
| VO₂max | 2041 | 0.480 | 2.36 | <0.001 | 0.022 |
| PFT per kg lean mass | 2097 | 0.664 | 2.37 | <0.001 | 0.040 |
| CFT per kg lean mass | 2064 | 0.772 | 2.39 | <0.001 | 0.040 |
| Power per kg | 1883 | 0.076 | 2.43 | <0.001 | 0.012 |

**Supplemental Figure S1.** Incremental and independent explanatory value of phase angle for physical performance outcomes. Left panel shows the increase in model R² (ΔR²) after adding phase angle to models adjusted for DXA-derived fat-free mass index, percent body fat, age, and sex. Right panel shows the partial R² for phase angle in the fully adjusted models. Phase angle demonstrated the greatest independent contribution for lean-mass–normalized performance outcomes and moderate contributions for PFT and CFT, with minimal contribution for VO_2_max.
